# Integrated Blockchain-Deep Learning Approach for Analyzing the Electronic Health Records Recommender System

**DOI:** 10.3389/fpubh.2022.905265

**Published:** 2022-05-06

**Authors:** Eric Appiah Mantey, Conghua Zhou, S. R. Srividhya, Sanjiv Kumar Jain, B. Sundaravadivazhagan

**Affiliations:** ^1^School of Computer Science and Communication Engineering, Jiangsu University, Zhenjiang, China; ^2^Sathyabama Institute of Science and Technology, Chennai, India; ^3^Department of Computer Science, Medi-Caps University, Indore, India; ^4^Department of Information Technology, Faculty of Information Technology, University of Technology and Applied Sciences-Al, Mussanah, Oman

**Keywords:** electronic health records, blockchain, deep learning, integrated environment, hyperledger fabric

## Abstract

Blockchain is a recent revolutionary technology primarily associated with cryptocurrencies. It has many unique features including its acting as a decentralized, immutable, shared, and distributed ledger. Blockchain can store all types of data with better security. It avoids third-party intervention to ensure better security of the data. Deep learning is another booming field that is mostly used in computer applications. This work proposes an integrated environment of a blockchain-deep learning environment for analyzing the Electronic Health Records (EHR). The EHR is the medical documentation of a patient which can be shared among hospitals and other public health organizations. The proposed work enables a deep learning algorithm act as an agent to analyze the EHR data which is stored in the blockchain. This proposed integrated environment can alert the patients by means of a reminder for consultation, diet chart, etc. This work utilizes the deep learning approach to analyze the EHR, after which an alert will be sent to the patient's registered mobile number.

## 1. Introduction

In this modern period, many clinicians do not maintain the physical ledger of a patient's information, diagnostic details, and treatments. Instead, they keep health records electronically. These records hold the patient's complete information such as his history, allergies, treatment taken, etc. It can be viewed by clinicians, hospitals, and healthcare providers for any cause. These records require the utmost safety and security in order to prevent misuse by third parties or cyber attackers (1, 2). It contains confidential information about medication, symptoms, scan reports, and even biometrics. This work uses blockchain-based storage for Electronic Health Records (EHR) to provide privacy and security for the data. It is very crucial that EHR be maintained because it contains sensitive information (3, 4). There are some drawbacks in existing EHR maintenance which include:

1. Whether to trust an organization that stores EHR.
2. Hacking and medical data breaching.
3. Difficulty in retrieving the patient's history for treatment suggestion.
4. In case of emergencies, how to get the patient data, and if it can be trusted.

To overcome all these drawbacks, the work proposes a storage method using a blockchain-hyperledger fabric to store the patient data with enhanced security and privacy. The medical report of any patient cannot be accessed without their permission. Other users like doctors, healthcare providers should have a digital certificate from the government to join and access EHR in the blockchain network. Only doctors, healthcare providers, and clinicians would be able to perform CRUD operations in the block. The data from the block is retrieved using the hash value. Next, the retrieved patient's block is analyzed using Recurrent Neural Network-Long Short-Term Memory (RNN-LSTM). Then the performance of RNN-LSTM is compared with RNN-GRU. Once the patient's block is analyzed, an alert will be sent to the patient's registered mobile number.

### 1.1. Motivation

The Electronic Health Records contain very sensitive information, and the chances of cyber attackers misusing the reports are very high in the centralized storage environment. Furthermore, the patients track their reports for regular consultation, medication, diagnosis appointments, etc. The motivation of this proposed work is to analyze the patient's EHR witha deep learning mechanism stored in the Blockchain and to create an alert system for the patient.

### 1.2. Contribution

To incorporate the reliable Hyperledger fabric blockchain to store the medical records of the patient. It also analyzes the blocks(the patient's EHR) using the RNN-LSTM and RNN-GRU mechanism. An alert system is created for the patients welfare, for activities like an alert for the next consultation, medication, and for diagnosis. The behavior and efficiency of the RNN algorithm is analyzed. Asa result, a reliable Hyperledger fabric is utilized for storing the EHR. Those medical records are analyzed by the deep learning Recurrent Neural Network algorithm, and it creates an alert system to monitor the patients remotely.

### 1.3. Organization of the Paper

The rest of the paper is organized as follows: Section 2 describes related works. Section 3 explains materials and methods. Section 4 discusses the results. Finally, Section 5 concludes the work.

## 2. Related Work

Blockchain is a booming technology which has been incorporated in many areas like industry and academic scenarios (3, 5). The diet recommendation system was created for patients affected by heart disease and to avoid heart disease (6), while also checking for the family history. Kutia et al. (7), Plastiras and O'Sullivan (8), and Shabbir et al. (9) studied the impact of different parameters that influence the adapting to and use of the online health facilities in Ukraine and China, such asallergies, food preferences, age, blood pressure. It utilizes machine learning algorithms for the recommendation system. The authors (8) proposed a tailored recommendationfor patients based on different parameters, with reference to their health records using Artificial Intelligence. The study (10) proposes a literature survey of machine learning algorithms that are used in a recommendation system. Agapito et al. (11) proposed a diet organizer system, which can be utilized by both healthy people and patients with chronic diseases. The authors in Mani et al. (12) and Singhal et al. (13) utilized the blockchain, Hyperledger fabric storage using Inter Planetary File Storage (IPFS) protocol to save data in the blockchain. Wang et al. (14) showed how bigdata analytics can be utilized for the healthcare industry, with the pros and cons of the model. The use of predictive data mining algorithms to recommend healthy diets was proposed by Jaiswal (15). A data mining model was developed to provide users with information about healthy food habits and eating patterns, such as the number of calories burned, the amount of macronutrients consumed, and so on. Patients' dietary preferences are predicted by the patient diet recommendation system by taking into account their eating habits and body measurements. In spite of its effectiveness in predicting healthy diets for patients and nutritionists/doctors, the study is limited by its lack of a flexible model and minimally tailored solutions to fit the patient's needs. Argaw et al. (16) briefly explains that clinical documents and hospitals are very prone to hackers and cyber attackers. There are scalability issues as the data is stored in an on-chain database, which makes the system unstable. However, the author in Egala et al. (17) proposed a ring structure-based access control that ensures privacy. Alsufyani et al. (18) proposed a system to manage data intelligently in a cyberspace environment. Decentralized storage and access to records, as described in Singh et al. (19), are efficient ways to utilize the network's power and resources. In Peng et al. (20), the author employed high-end privacy-enhancing technologies, such as homomorphic encryption, that prevent vulnerabilities due to the processing of data while it is encrypted. To ensure nodes did not engage in malicious behavior, the Agrawal and Jain (21) used zero-knowledge proof and proof authority consensus for mutual authentication as a privacy enhancement technique. There is no doubt that blockchain technology provides a cryptographic solution to the security issue, but there are challenges such as privacy, scalability, and interoperability. Large medical data centers have experienced record breach episodes in the past two decades, which have left medical companies with additional challenges (21). When blockchain technology was newly invented, MedRec (22) became the first recommendation system for an electronic patient record management system that would be made possible with blockchain technology. Ether blockchains are capable of backing up detailed accessibility data. Healthcare providers maintain third-party databases, not the blockchain. This means that the records can still be hacked or misused. A healthcare management system as in Ivan (23) encrypts patient keys as data is recorded on the blockchain. Researchers and hospitals decrypt the data with a patient's public key after receiving consent from the patient. In contrast, we empower our patients to control their own data, so they are the only ones who have access to them.

## 3. Materials and Methods

### 3.1. Hyperledger Fabric

**Figure 1:**
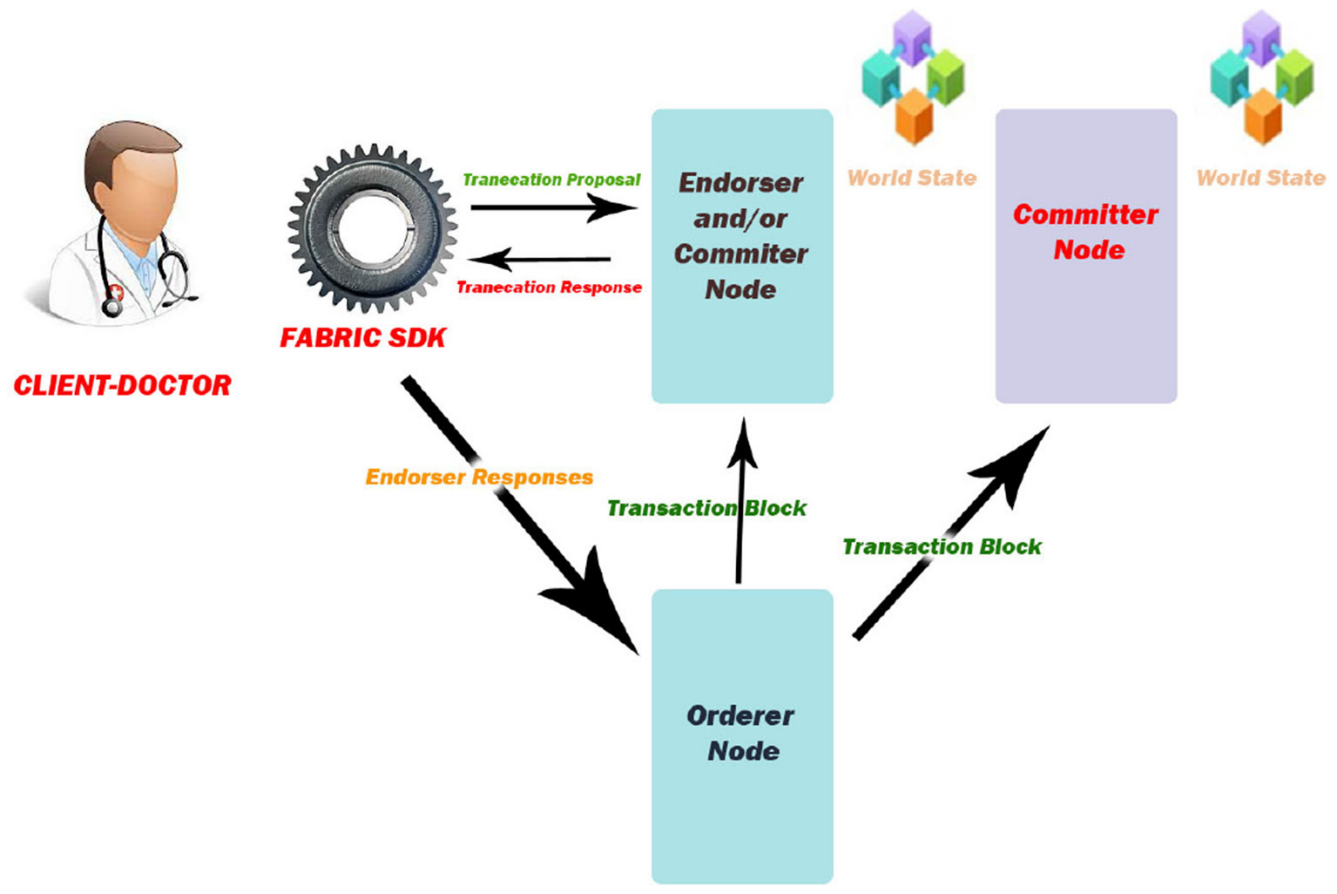
Architecture of hyperledger fabric.

Hyperledger fabric is an open-source platform which builds the distributed ledger, with a plug-and-play architecture (modular) that facilitates a high level of security, privacy, and confidentiality of the data. The architecture of Hyperledger fabric is shown in Figure 1. The client capitulates the transaction pool through the fabric SDK to the endorser. Endorsing peers verify and execute the transaction, and generate the read and write sets. The response is then sent back to the client. All replies from peers are collected by the client, and they are sent to the “orderer.” In this case, all transactions are ordered by the orderer in ascending order and then formed into a block. Each committer validates this block and adds a new block to their own copy of the ledger as a result. The fabric consists of four components, namely:

1. Membership Service Provider (MSP)
2. Client
3. Orderer
4. Peer

Membership Service Provider is the main component of Fabric which defines a rule to allow members in after authentication and verification. It manages the User ID and the authenticity of the client. The client is the person who initiates the transaction proposal. All transactions have to be written in the shared ledger in blockchain in a consistent order. In order for updates to the world state to be valid, the order in which they are made must be determined.

### 3.2. RNN-Long Short-Term Memory

**Figure 2:**
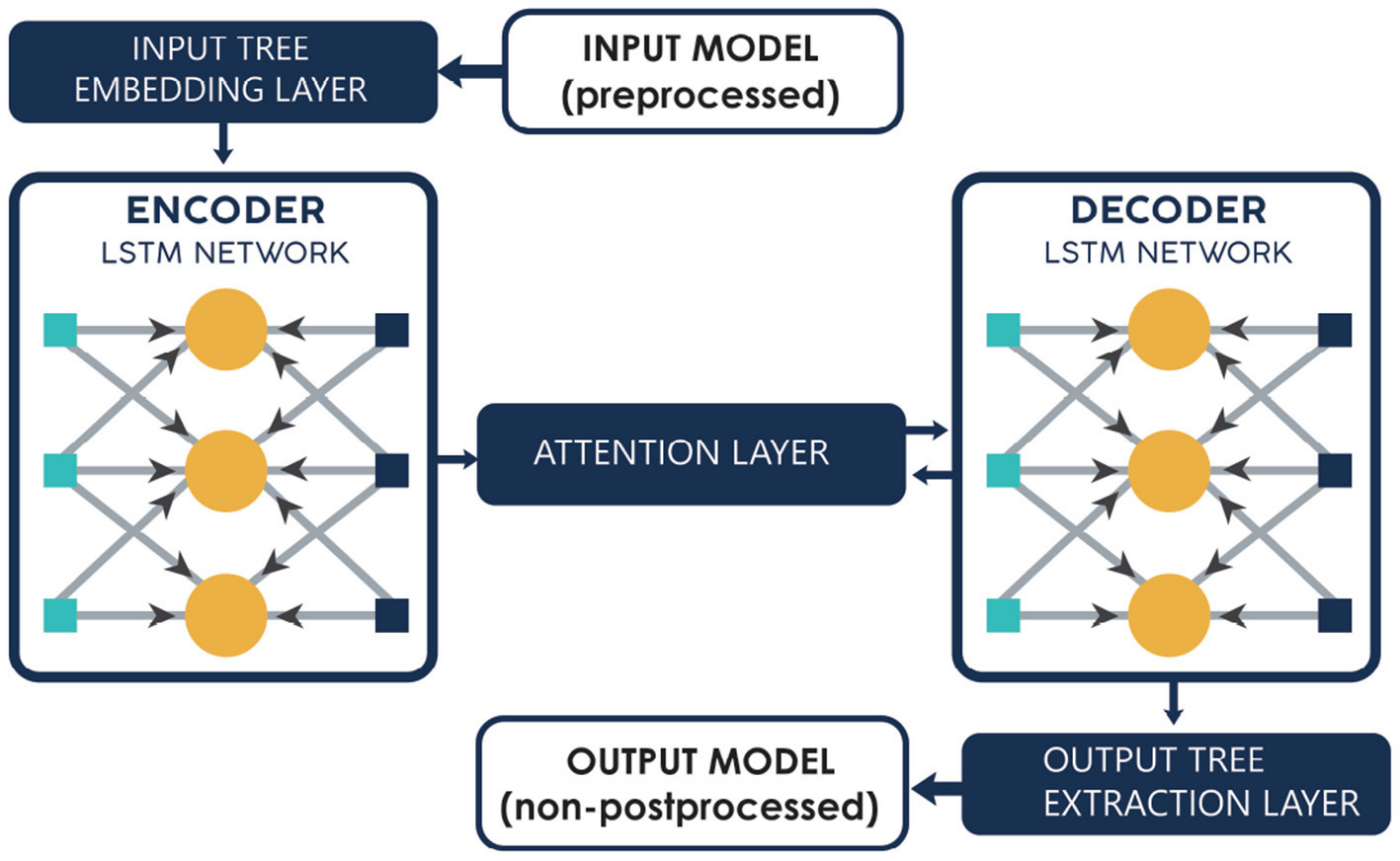
Architecture of the LSTM RNN.

A Recurrent Neural Network is a special kind of deep learning where the output of the previous step will be the input of the subsequent step. LSTM is a special type of Recurrent Neural Network which enables the system to learn the long-term dependencies of data. This type of learning is achieved by the repeated module of the LSTM, which has a combination of 4 different layers connected to each other. Figure 2 explains the method, and the three-layeredarchitecture where the embedding layer gives the pre-processed data to the Encoder-LSTM network. It then passes to the attention layer that acts as the intermediate layer, where it facilitates the decoder to pay more attention to the specific parts of the fixed-size vectors.

#### 3.2.1. RNN-GRU

The Gated Recurrent Unit (GRU) is another type of Recurrent Neural Network. It is very similar to the Long-Short-Term Memory RNN.

#### 3.2.2. System Model

This work utilizes the blockchain to store all the EHR of the patients, which cannot be accessed by third parties or cyber attackers, because of its high security and privacy of medical data. Hyperledger Fabric can be used to save these types of medical data. It uses Inter Planetary File System (IPFS) which provides a solution for the file storage problems. IPFS can store and retrieve large files efficiently. Health records are encrypted using symmetric key cryptography to ensure privacy. To protect the record, it is stored in an encrypted format on an IPFS server under the appropriate supervisory authority. To access the patient's record, an entity must first be given the authorization to do so.

1. A private key is used to decrypt the record.
2. RSA key pair public key and symmetric key are used to encrypt the key.

Access to a health record may be removed if:

1. The symmetric key is decrypted by the private key associated with the EHR owner.
2. The symmetric key is used to decrypt the EHR.
3. A new symmetric key is used to re-encrypt the record.
4. Encryption of the symmetric key is completed using the public keys of all authorized users.

**Figure 3:**
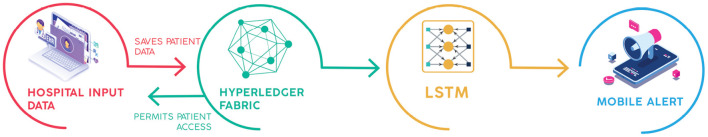
Model of the system.

Figure 3 describes the model of the system it takes the Input (EHR) from clinicians, doctors, and healthcare providers which is stored in the Hyperledger Fabric using the protocol Inter Planetary File System (IPFS) using Algorithm 1, where from the data is retrieved by a deep learning agent for analyzing the EHR, after analyzing the data in EHR the RNN-LSTM will send the alert for several activities like doctor's consultation, medication, and diagnosis schedule to the patient's registered android device, this was explained in the Algorithm 2.

**Algorithm 1:**
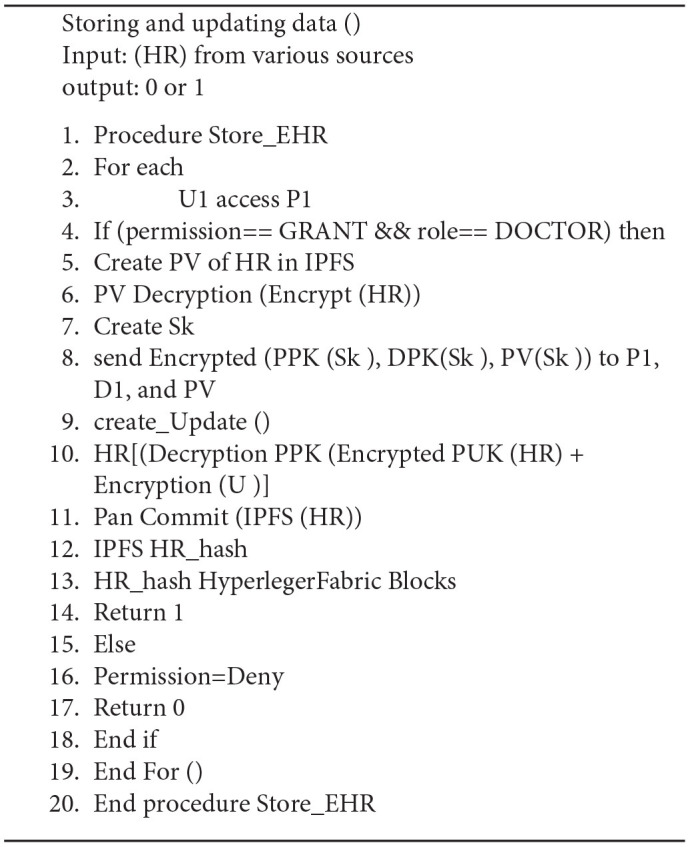
Creating and updating health records in Hyperledger blockchain.

**Algorithm 2:**
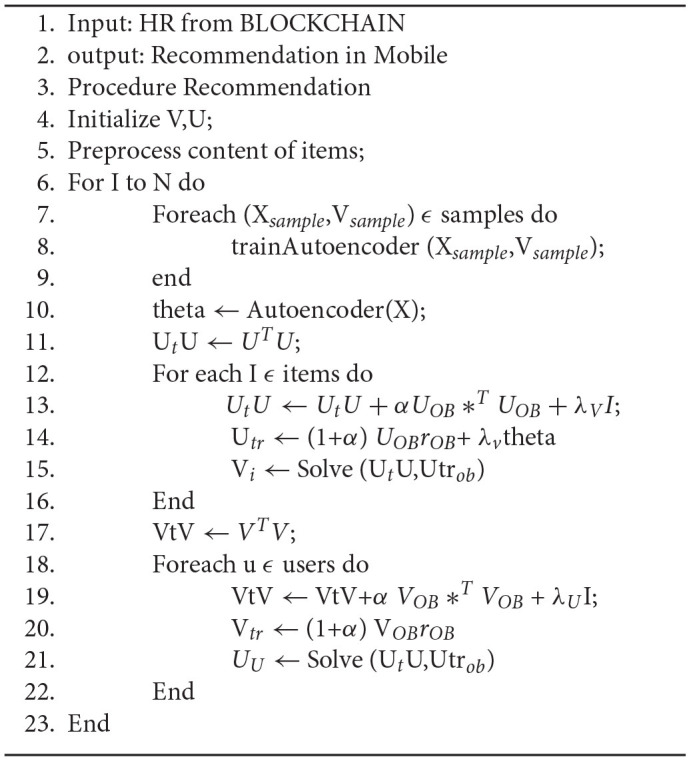
Creating an alert for health records in Hyperledger blockchain Alert ().

This proposed work uses nine different features from EHR to provide an effective alert system. These features are listed in Table 1 above. Considering all these features, we can design an efficient alert system, through whichregular monitoring of thepatient can be done.

**Table 1:** Reference summary.

**S.no**	**Health information system**	**Challenges**	**Area to be concentrated**
1	Electronic health records [21]	Interoperability, access control, data integrity	Shared decision making
2	Electronic health records [22]	Interoperability	Health data recording, storing, and sharing Access control
3	Electronic health records [23]	Interoperability	Sharing of healthcare information for clinical and research purposes Access control
4	Electronic health records [24]	Data integrity, access control	Sharing healthcare data between health institutions
5	Electronic health records [25]	Data integrity	Sharing healthcare data between health institutions
6	Electronic health records [26]	Access control, interoperability, data integrity	Sharing healthcare data for clinical and research purposes
7	Electronic health records [27]	Access control, interoperability	Sharing healthcare data for clinical and research purposes
8	Electronic health records [28]	Access control, interoperability and administrative	Sharing healthcare (health record) information for clinical, research[economic] purposes.
9	Electronic health records [29]	Access control, data integrity, interoperability	Patients collection, archiving and sharing of healthcare data for clinical purposes
10	Electronic health records [30]	Patient data management and storage	Environment Access control, data integrity, data provenance

### 3.3. Data Preprocessing

Data pre-processing is an essential task to be carried out. It helps to achieve the maximized efficiency of the model. At this stage, the dataset is pre-processed by four important steps which include:

1. Data cleaning
2. Data integration
3. Data transformation
4. Data reduction

These four steps have to be followed sequentially to get the required pre-processed data to train the model. At first, the dataset is cleansed by assigning missing values, null values, eliminating the nosy data, resolving the inconsistency,and removing outliers. The cleansed data is then used for data integration, which refers to the merging of data from multiple sources into a single larger data store, such as a data warehouse. Inthe data transformation step, wetransform the value, structure, or format of quality data into alternative forms, according to some strategies such as generalization, normalization, attribute selection and aggregation. Finally, the data reduction is carried out to find the appropriate dataset model proposed to achieve the maximum efficiency.

### 3.4. Evaluation Metrics

Many evaluation metrics are used to evaluate the performance of our model


(1) $$\begin{array}{r} Accuracy=\left(AZ+AN\right)/\left(AZ+AN+CZ+CN\right) \end{array}$$

(2) $$\begin{array}{r} Precision=AZ/\left(AZ+CZ\right) \end{array}$$

The objective of precision is to examine the True Positive (AZ) units in connection with False Positive (CZ) units.

The objective of recall is to examine True Positive (AZ) units in connection to False Negative (CN) units that are not classified. The arithmetic arrangement of recall is stated in Equation below:


(3) $$\begin{array}{r} Recall=AZ/\left(AZ+CN\right) \end{array}$$

Sometimes, the assessment of performance may not be very efficient with recall and accuracy. For example, if a mining algorithm has high precision but low recall, then another algorithm is needed. Then comes the question of which algorithm is more effective. This challenge is solved utilizing F1-measure that gives a mean recall and precision. F1-measure can be calculated as follows:


(4) $$\begin{array}{r} F1score=2*\left(Precision*Recall\right)/\left(Presiion+Recall\right) \end{array}$$

### 3.5. Experiments and Results

In the proposed model, the personal alert system is designed for patients registered mobile numbers. The alert is created for certain activities like the next consultation date, medication, diet specification, and diagnosis date. Initially, the patient's EHR is stored in the Blockchain using IPFS protocol. The data saved in the blockchain will be more secure and distributed. The data that is saved in the blockchain is retrieved by the deep learning mechanism, Long Short-Term Memory for analyzing the data. After analyzing the patient EHR, a tailored alert system is triggered to the registered mobile number.

The proposed work uses nine features from the dataset, the features are ID, age, gender, disease, weight, consultation date, medication, diagnosis, and diet specification. The taxonomical analysis is done with several featuresto give accurate results.

The analysis of the EHR is done using the deep learning techniques Long-Short Term Memory (LSTM) and Gated Recurrent Unit (GRU) which are Recurrent Neural Network techniques. Table 2 shows the list of abbreviation and Table 3 shows the features used in the model. Therefore, the comparison between the two integrated blockchain and Recurrent Neural Networks in analysing the EHR is given in Table 4, in terms of precision, recall, and F1 score.

**Table 2:** List of abbreviations.

HR	Health Record
P1	Patient
D1	Doctor
U1	User
PPK	Patients Private key
PUK	Patients Public Key
Sk	Session Key
DPK	Doctors Private key
PV	Patient View of data

**Table 3:** Features used in the model.

**S. No**	**Features**	**Type of feature values**
1	Patient_ID	Numeric
2	Patient_age	Numeric
3	Patient_Gender	Categorical
4	Patient_Weight	Numeric
5	Patient disease	Categorical
6	Medication chart	Text
7	Next Appointment date	Date
8	Next diagnosis date	Date
9	Diet specification	Text

**Table 4:** Report of precision, recall, and F1 scores.

**Parameters**	**Label**	**Integrated** **hyperledger** **Fabric-LSTM**	**Integrated** **hyperledger** **Fabric-GRU**
Precision	Allowed Not allowed	0.9876 0.8943	0.9654 0.9923
Recall	Allowed Not allowed	0.9946 0.7231	0.9986 0.3743
F1 Scores	Allowed Not allowed	0.9921 0.8276	0.9723 0.5523

**Figure 4:**
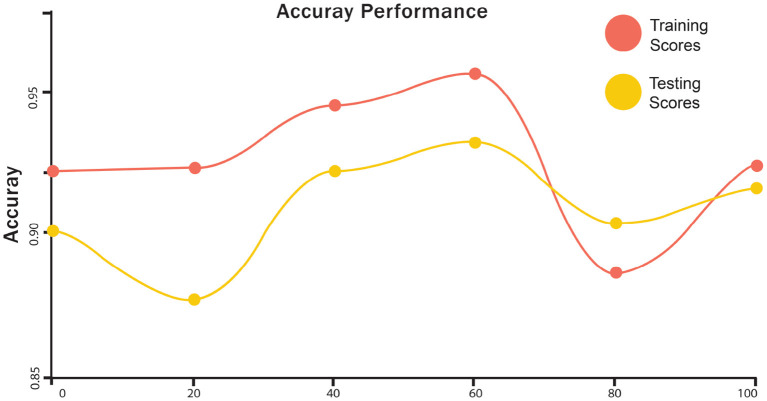
Accuracy performance of training and the testing score of LSTM.

**Figure 5:**
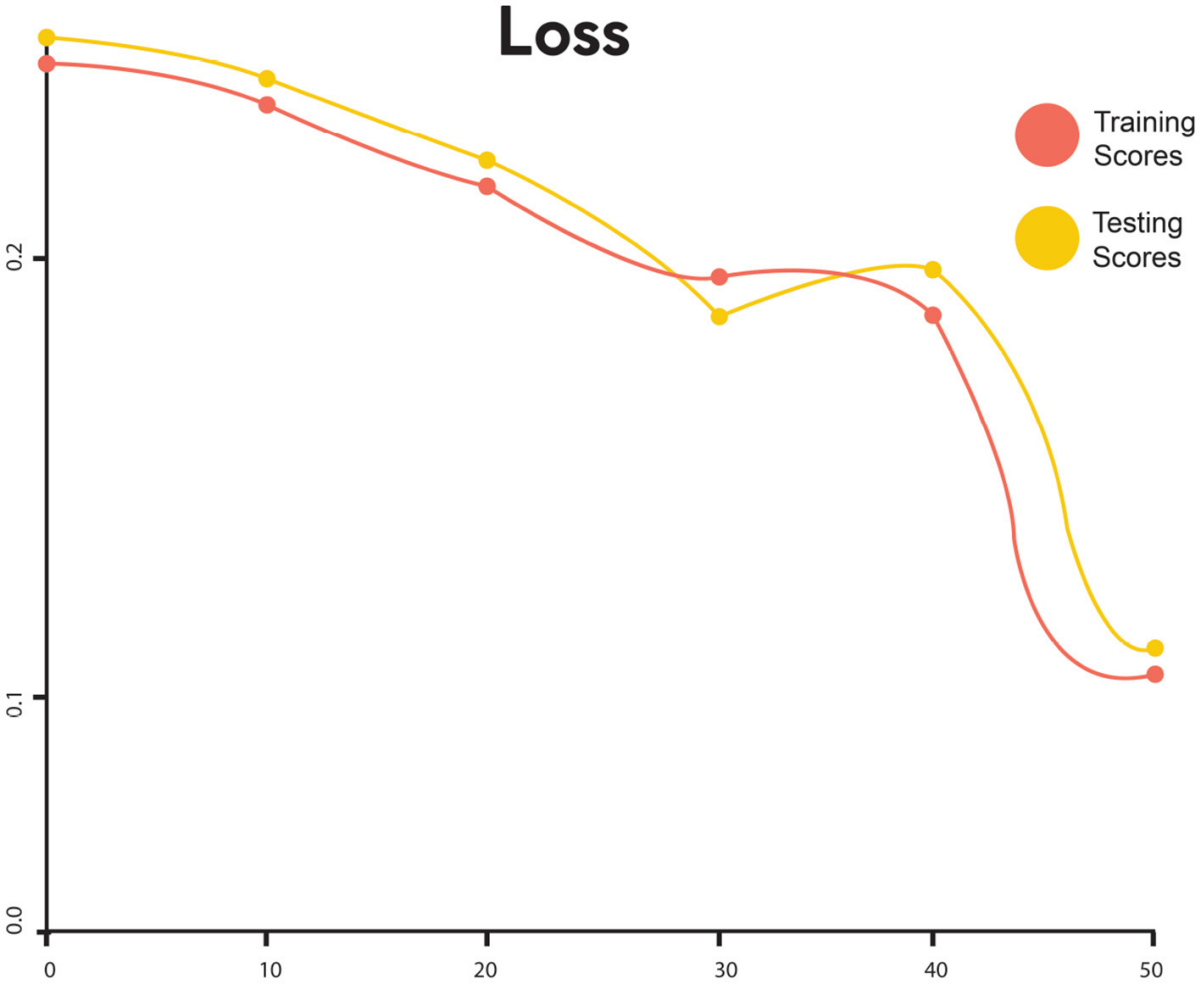
Loss performance of training and the testing score of LSTM.

**Figure 6:**
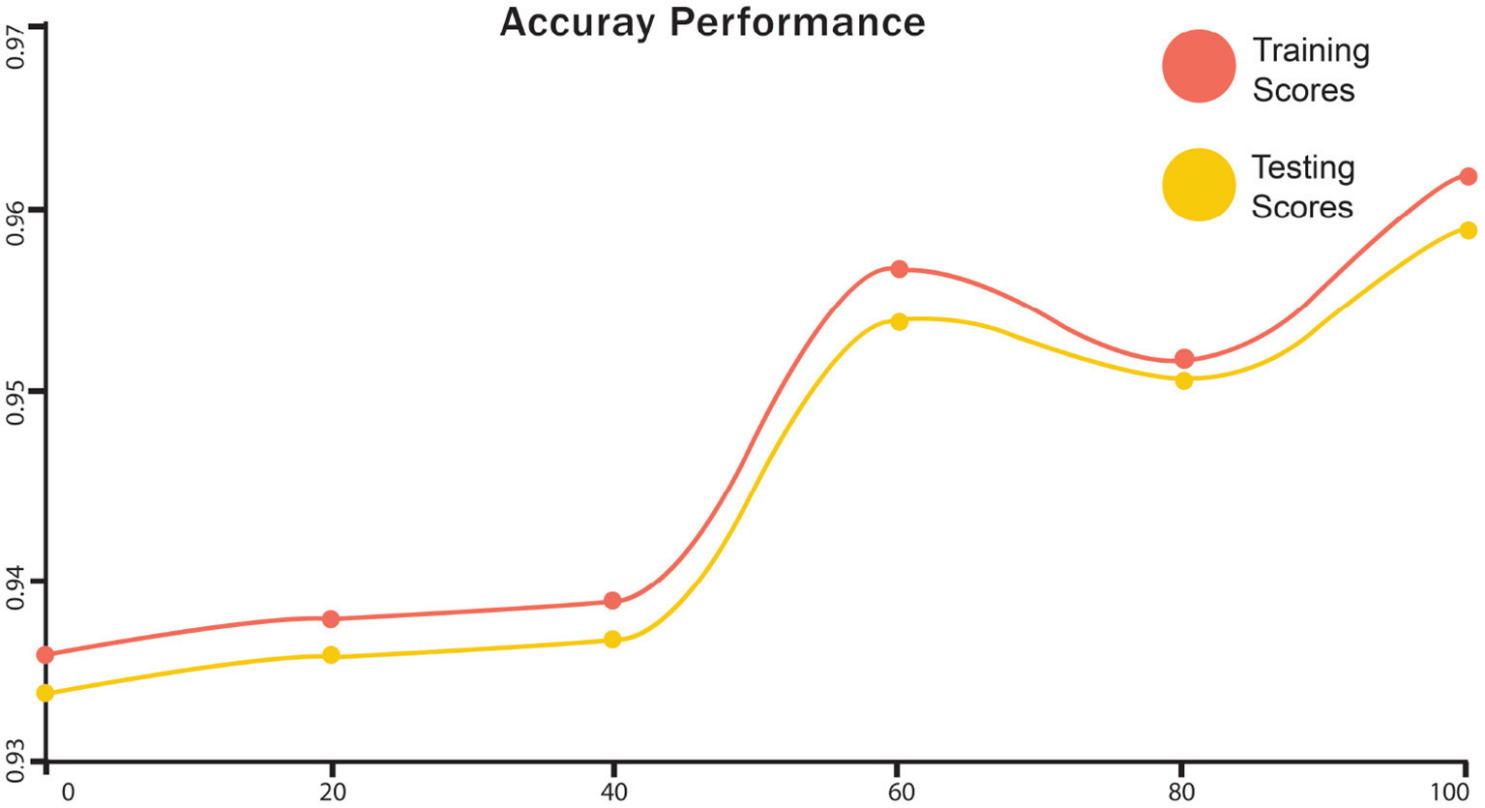
Accuracy performance of training and testing score of GRU.

**Figure 7:**
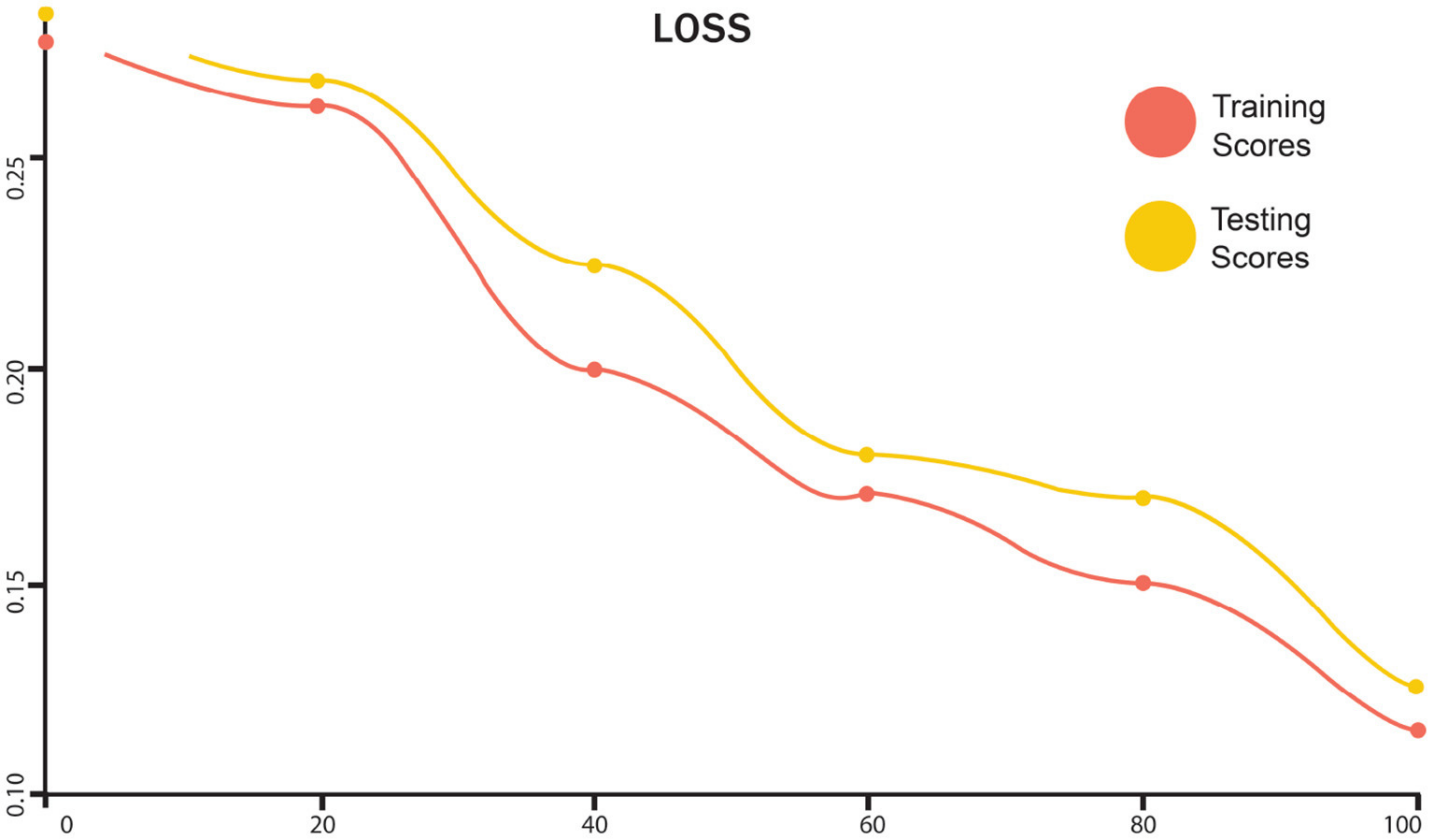
Loss performance of training and testing score of GRU.

The training and testing scores for LSTM are shown in Figure 4. In the graph, the red line represents the training curve and the yellow line represents the testing curve. Training curves start from 93.6% and after 50 epochs, they reach 95%. For the testing curve, it starts at 94% and goes to 97.4%, then goes down to 96%. Training and testing losses for LSTM are shown in Figure 5. The training loss is represented in red, while the testing loss is displayed in yellow. The training loss begins at 0.27 and reduces to 0.128. The testing loss begins at 0.23 and decreases to 0.075. Figure 6 shows the scores obtained during the training and testing for GRU, while Figure 7 shows the losses obtained during the training and testing for GRU. The red curve in Figure 5 represents the training curve. It starts at 93% and goes up to 97% testing and training scores for GRU. Similarly, the yellow curve representing the LSTM's testing score starts at 94% and goes up to 97%. Figure 7 shows the training loss starts at 0.3 and decreases after 50 iterations. It reaches 0.1 after that. Likewise, the testing loss starts at 0.2 and decreases until it reaches 0.05.

**Figure 8:**
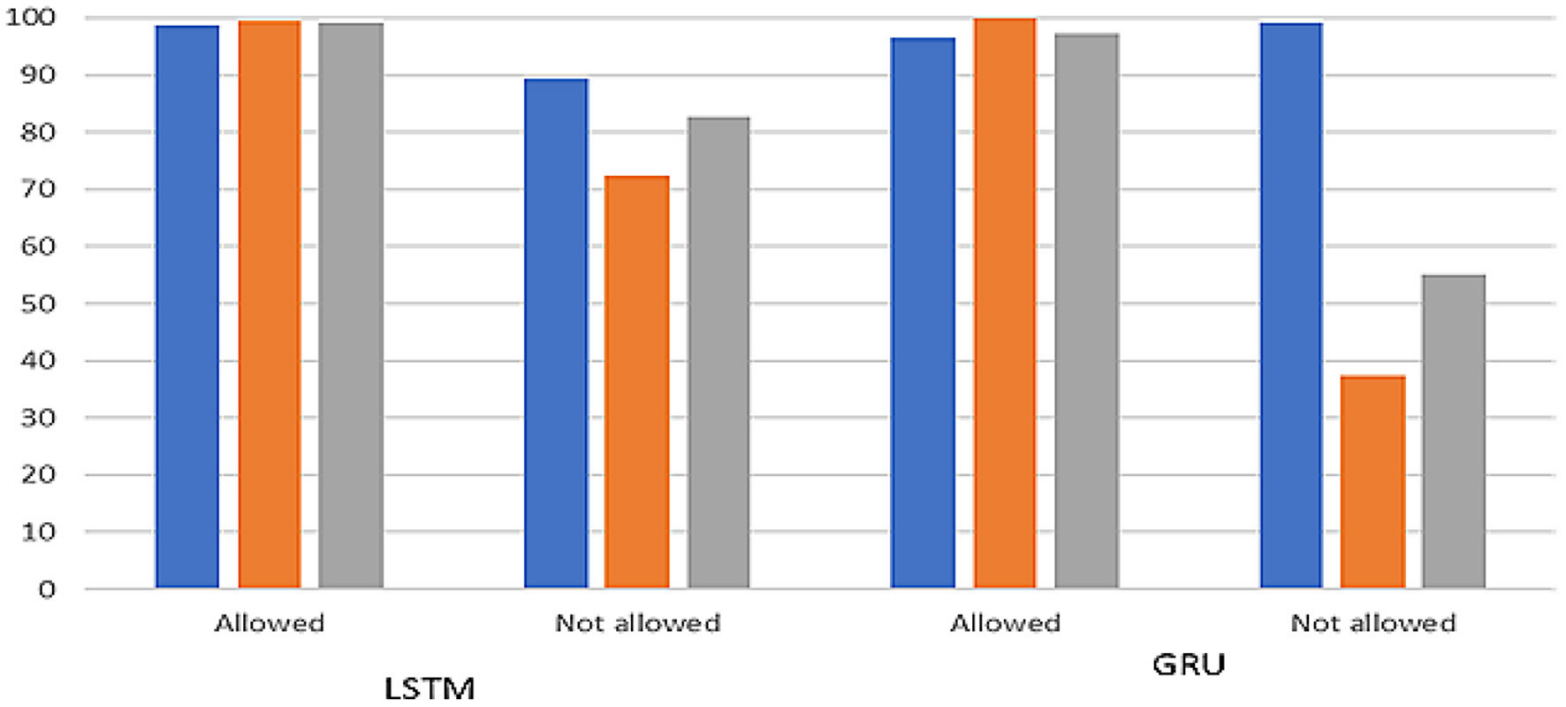
Classification report of LSTM and GRU.

Table 2 shows that the LSTM model outperforms another modelin terms of recall, precision, and F1 score. The Allowed LSTM had 98% precision, 99% recall, and F1 measure scores, respectively. The Not Allowed class had 87% precision,77% recall, and 81% F1 measure score, respectively. The other model Integrated Blockchain-GRU also performs well but not as well as LSTM. Finally, the result of the model is shown for both Allowed and Not Allowed classes in Figure 8.

## 4. Conclusion

The study proposes an integrated blockchain-RNN algorithm for storing the EHR in the Hyperledger Fabric using the protocol Inter Planetary File System. The stored EHR is analyzed with the deep learning mechanism, Recurrent Neural Network algorithms, namely Long-short-Term Memory and Gated Recurrent Units. The integrated model gives an alert to the registered mobile number of patients regarding the consultation reminder, diagnosis alert, medication, diet chart specification. It uses 9 features, i.e., age, sex, weight, disease, medication chart, appointment date, diagnosis date, and diet specification. This proposed work focuses on an integrated model to automate the alert system for various activities of the patient. Finally, experimental results show that the LSTM outperforms the other models in terms of precision, recall, and F1 score. This work is practically possible but the maintenance cost is more when compared to the traditional model. In the Future, the alert system can be improved by collaborating with the calendar application in android mobile with fitness applications and to provide a solution for a cost-efficient model.

## Data Availability Statement

The original contributions presented in the study are included in the article/Supplementary Material, further inquiries can be directed to the corresponding author.

## Author Contributions

All authors listed have made a substantial, direct, and intellectual contribution to the work and approved it for publication.

## Funding

This article was supported by the key research and development plan (social development) projects BE2016630 and BE2017628 of Jiangsu province, the scientific research project Z201603 of Wuxi health and family planning commission.

## Conflict of Interest

The authors declare that the research was conducted in the absence of any commercial or financial relationships that could be construed as a potential conflict of interest.

## Publisher's Note

All claims expressed in this article are solely those of the authors and do not necessarily represent those of their affiliated organizations, or those of the publisher, the editors and the reviewers. Any product that may be evaluated in this article, or claim that may be made by its manufacturer, is not guaranteed or endorsed by the publisher.
