# Supplementary figures and images for: Integrated Blockchain-Deep Learning Approach for Analyzing the Electronic Health Records Recommender System
Source: Front Public Health. 2022 May 6;10:905265. doi: 10.3389/fpubh.2022.905265 (PMC9122032; doi:10.3389/fpubh.2022.905265)

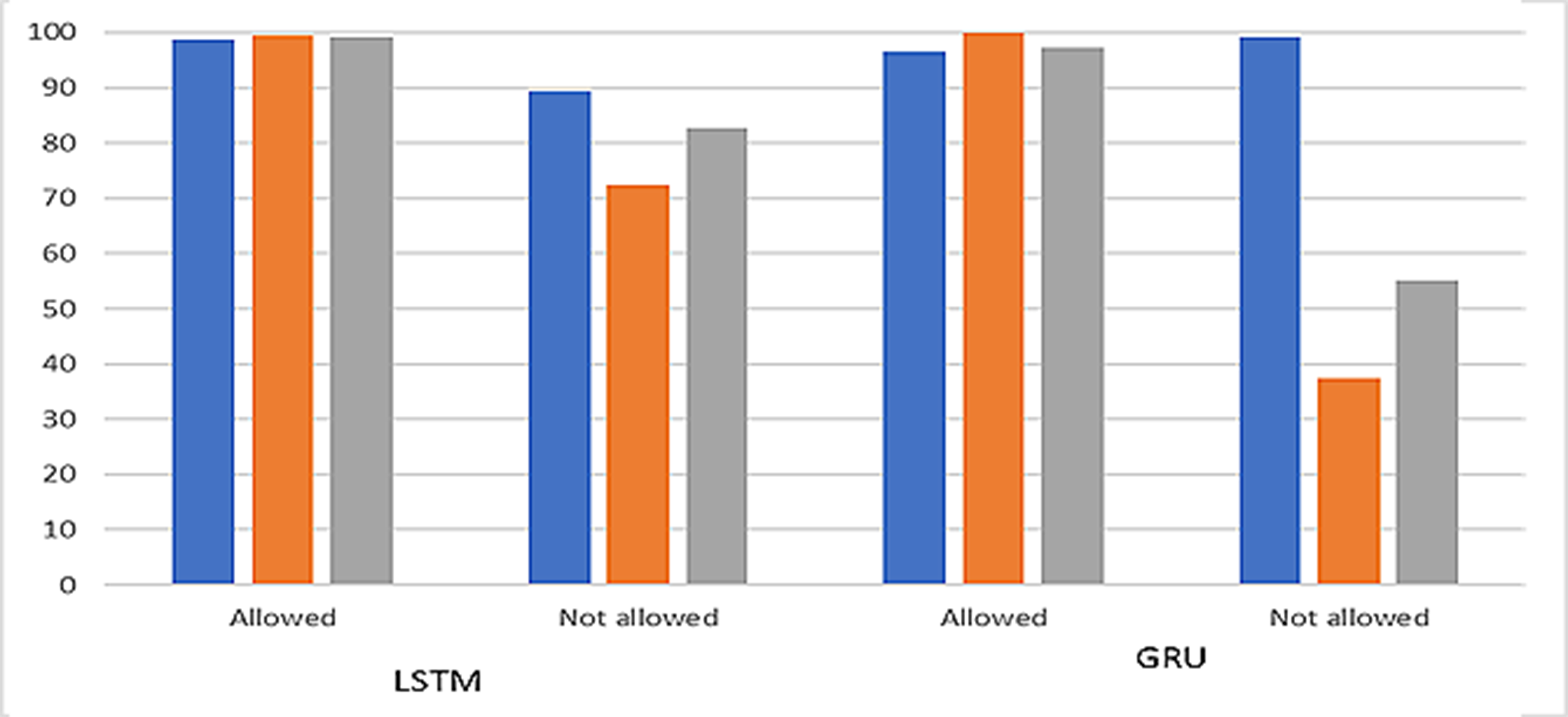

Supplement: Supplementary file 2 [file Image_2.PNG]
